# Protein Z/protein Z-dependent protease inhibitor system in loco in human gastric cancer

**DOI:** 10.1007/s00277-013-1941-8

**Published:** 2013-10-26

**Authors:** Ewa Sierko, Marek Z. Wojtukiewicz, Lech Zimnoch, Piotr Tokajuk, Krystyna Ostrowska-Cichocka, Walter Kisiel

**Affiliations:** 1 Department of Oncology, Medical University, 12 Ogrodowa St., Bialystok, Poland; 2 Comprehensive Cancer Center, Bialystok, Poland; 3 Department of Clinical Pathomorphology, Medical University, Bialystok, Poland; 4 Department of Pathology, School of Medicine, University of New Mexico, Albuquerque, NM USA

**Keywords:** Gastric cancer, Protein Z, Protein Z-dependent protease inhibitor, Coagulation inhibitor, Factor X

## Abstract

In gastric cancer, hemostatic system components contribute to cancer progression, as activation of factor X (FX) was observed. The protein Z (PZ)/protein Z-dependent protease inhibitor (ZPI) complex inhibits factor Xa proteolytic activity. The purpose of this study was to determine the distribution of ZPI and PZ in relation to FX, and prothrombin fragment (F1 + 2), a standard marker for blood coagulation activation, in human gastric cancer tissue. ABC procedures and a double staining method employed polyclonal antibodies against PZ, FX, and F1 + 2 and a monoclonal antibody against ZPI. In situ hybridization (ISH) methods employed biotin-labeled 25-nucleotide single-stranded DNA probes directed to either PZ or ZPI mRNAs. FX and components of PZ/ZPI coagulation inhibitory system were observed in cancer cells. F1 + 2 was observed in gastric cancer cells as well. Double staining studies revealed FX/PZ, FX/ZPI, and PZ/ZPI co-localization on gastric cancer cells. ISH studies demonstrated the presence of PZ mRNA and ZPI mRNA in gastric cancer cells indicating induced synthesis of these proteins. The co-localization of PZ/ZPI and FX in gastric cancer cells indicates in loco that these proteins may play a role in anticoagulant events at the tumor tissue.

## Introduction

Gastric cancer is one of the leading causes of cancer-related death in Europe [1]. Thromboembolism complicates the course of gastric cancer in approximately 15 % of these cases [2]. These include, inter alia, deep vein thrombosis, portal vein thrombosis, Budd–Chiari syndrome, nonbacterial thrombotic endocarditis, pulmonary thromboembolism, as well as disseminated intravascular coagulation [2]. Thromboembolic complications may be the first manifestation of gastric cancer or occur during the treatment (e.g., surgery) or disease progression [2]. Evidence indicates that blood coagulation activation proceeds not exclusively in blood vessels but also in gastric cancer tissue [2]. Tumor-specific activation of factor X (FX) in cancer patients has been reported, which suggests an important role for this stage of coagulation activation in hemostatic system alterations during malignant tumor progression [3, 4].

That hemostatic system components contribute to cancer growth and dissemination has been well-documented [4–7]. Factor Xa activity, and consequently the rate of thrombin generation, is precisely controlled by several inhibitory mechanisms such as tissue factor pathway inhibitor (TFPI), antithrombin, and the protein C (PC) system [8]. A previous study revealed that while FX was present in association with gastric cancer cells, TFPI was not observed in this localization [2]. Furthermore, PC was detected in association with gastric cancer cells and endothelial cells (ECs), but the study failed to demonstrate the presence of protein S on these cells [2]. The abovementioned mechanisms suggest inadequate blood coagulation regulation at the tumor tissue. However, another mechanism of direct FXa inhibition, derived from the concerted activity of protein Z (PZ) and the protein Z-dependent protease inhibitor (ZPI), has been reported [9–12]. In this process, PZ, lacking any enzymatic function, serves as a co-factor in the reaction of FXa inhibition via ZPI [10, 11, 13, 14]. The reaction rate is increased by PZ more than 1,000-fold, which efficiently facilitates reduced thrombin generation [10]. The PZ/ZPI complex limits the coagulation response prior to the formation of the prothrombinase complex. Protein Z and ZPI circulate in plasma forming a complex. PZ interacts with FXa in the presence of membrane phospholipids, which kinetically optimizes the inhibition of membrane-associated FXa by ZPI [15–17]. ZPI can be also activated by glycosaminoglycans on the ECs surface and inhibits FXa that escapes from procoagulant phospholipids [18]. Appropriate inhibition of FXa via the PZ/ZPI system requires the presence of the inhibitory proteins and their precise co-localization at the tumor site. In this regard, there is scant data concerning PZ/ZPI inhibition of FX in gastric cancer tissue.

The aim of this study was to analyze the solid-phase interaction between expression of FX and PZ/ZPI in relation to blood coagulation activation, indicated by the potential presence of prothrombin fragment F1 + 2 in human gastric cancer. Furthermore, in order to determine the potential origin of PZ and ZPI, we also examined the expression of mRNAs encoding PZ and ZPI in these tumor sections.

## Materials and methods

Studies were performed on gastric cancer sections obtained during surgery of previously untreated gastric cancer patients. Normal gastric tissues, derived from neoplasm-free resection margins, were also obtained during surgery for comparison. The study protocol was approved by the local ethics committee of the Medical University in Bialystok, Poland. Informed consent was obtained from 15 patients. Histopathologic examination of tissue fragments revealed adenocarcinomas G2 in 11 cases and G3 in 4 cases. Clinical stage of the disease was assessed as T2-3N0M0. The tissues were fixed in buffered 4 % formalin. Immunohistochemical (IHC) procedures were performed according to the avidin–biotin complex technique (ABC) using reagents (Vectastain Kits, Vector Laboratories, Burlingame, CA, USA) described in detail elsewhere [19]. A semiquantitative analysis of protein expression, based on the percentage of positive staining cancer cells and the intensity of the staining, was performed exclusively for cancer cells as well [20, 21]. Immunoreactive score values ranging between 1 and 4 were interpreted as weak, 5 and 8 medium, and 9 and12 strong protein expression, respectively [20, 21]. A monospecific polyclonal antibody against homogeneous plasma-derived human PZ was prepared in rabbits, and then purified from immune sera by protein A-Sepharose chromatography [22]. Specific mouse monoclonal anti-human ZPI IgM (4249.2) was a generous gift from Dr. George J. Broze Jr (Division of Hematology, Barnes-Jewish Hospital, St. Louis, MO, USA) [23]. Polyclonal antibodies directed to coagulation FX and F1 + 2 were generously provided by Dr. David Stump (Genentech, South San Francisco, California) [24]. In the control specimens, primary antibody was omitted from the procedure. In the ABC IHC procedure, antigen staining was detected by the dark brown reaction product.

The Dako EnVision^TM^ (Dako, Carpinteria CA, USA) protocol, provided by the manufacturer utilizing the commercially available Dako EnVision^TM^ kit (Dako, Carpinteria CA, USA), was introduced in IHC double staining studies. Antibodies against FX, PZ, and ZPI, which were applied in the double staining procedure, were the same as those used in the ABC IHC study.

The IHC double staining study was aimed to directly compare the precise localization of the following:

- FX and PZ (FX was visualized as dark brown staining, whereas PZ as red reaction product)
- FX and ZPI (factor X was visualized as red staining, whereas ZPI as dark brown reaction product)
- PZ and ZPI (PZ was visualized as red reaction product, while ZPI as dark brown staining)

The in situ hybridization (ISH) protocol employed biotin-labeled 25-nucleotide single-stranded DNA probes: (probe sequence: 5′ biotin-CGTCATACCGCATGTGCACATGGAC-biotin 3′) specific for PZ mRNA [22], (probe sequence: 5′ biotin-GCCATCGTGCCTCATGGAGATCTTT -biotin 3′) specific for ZPI mRNA [23]. The probes were synthesized by Sigma-Aldrich, Poznan, Poland, and the ISH protocol of R&D Systems (R&D Systems, Minneapolis, MN, USA) was followed. Hybrids were detected using rabbit anti-biotin monoclonal antibodies according to the IHC ABC technique associated with ImmunoMax amplification procedure, which was described in detail elsewhere [25, 26]. Negative controls were based on hybridization without addition of the molecular probe and incubation of slides in RNase A solution (R&D Systems, Minneapolis, MN, USA) before hybridization. Reaction products appeared as dark brown staining.

Two independent observers provided visual assessment of the protein and mRNA expressions in ten consecutive high-power fields. The results of IHC and ISH studies of gastric cancer tissues were compared with the results obtained in respective normal gastric tissues, which were processed simultaneously.

## Results

**Fig. 1:**
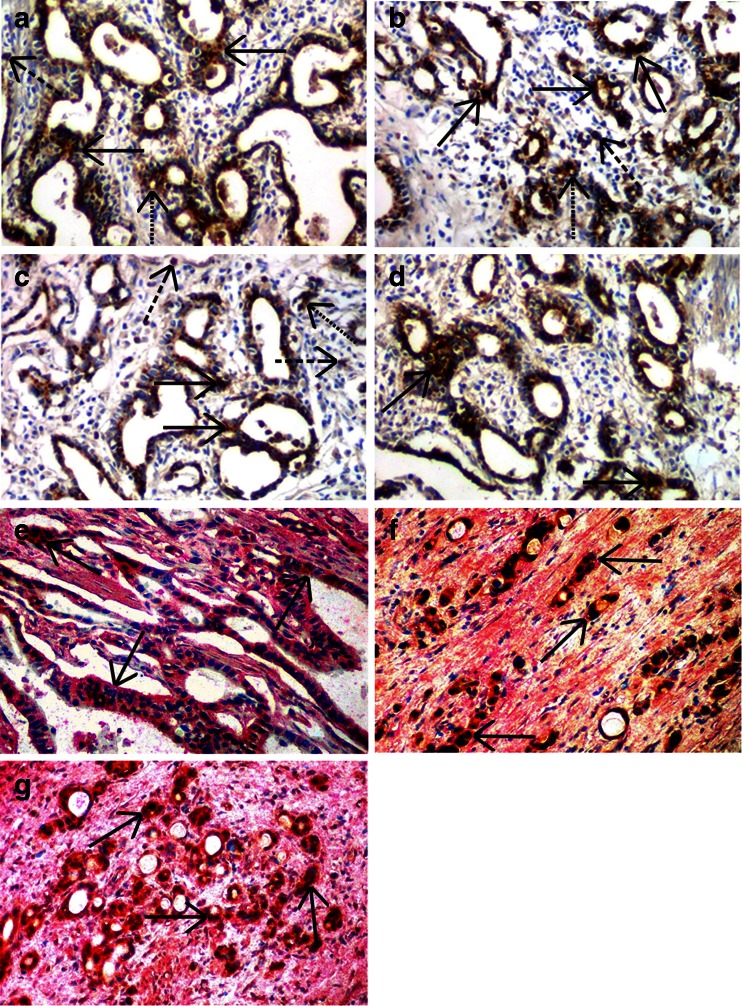
Specific staining (*brown* reaction product) by the immunohistochemical ABC peroxidase technique using polyclonal antibodies against coagulation factor X-FX (**a**), against protein Z-PZ (**b**) and against prothrombin fragment F1 + 2 (**d**), as well as a monoclonal antibody against protein Z-dependent protease inhibitor (*ZPI*) (**c**). *Solid arrows* indicate staining of tumor cell bodies in gastric cells, *dotted arrows* of endothelial cells, whereas *dashed arrows* of tumor-associated macrophages. Hematoxylin counterstain, original magnification ×100. Specific double staining Dako EnVision^TM^ technique (**e**, **f**, **g**) using polyclonal antibodies against FX and PZ as well as the monoclonal antibody directed to ZPI. **e** FX and ZPI (FX was visualized as *dark brown* reaction product, whereas PZ as *red* staining), **f** FX and PZ (factor X was visualized as *red* reaction product, whereas ZPI as *dark brown* staining), **g** PZ and ZPI (PZ was visualized as *red* staining, while ZPI as *dark brown* reaction product). The two colors are overlapping indicating co-expression of both proteins in gastric cancer cells (indicated by *arrows*). Hematoxylin counterstain, original magnification ×100

In gastric cancer, FX and components of the PZ/ZPI coagulation inhibitory system were observed in association with cancer cell bodies (Fig. 1a, b, c; Table 1). Furthermore, F1 + 2, indirectly indicating extravascular activation of blood coagulation at the tumor site [24], was also detected in association with cancer cells (Fig. 1d; Table 1).

**Table 1:** Median immunoreactive score (IRS) values of cancer cell expression of coagulation factor X (FX), protein Z (PZ), protein Z-dependent protease inhibitor (ZPI) and prothrombin fragment F1 + 2 (F1 + 2) in gastric cancer. ABC immunohistochemical staining

	Factor X	ZP	ZPI	F1 + 2
Median IRS	9.0	8.0	8.0	6.5

Additionally, both PZ and ZPI were observed in association with ECs of small blood vessels supplying the gastric tumor, and in tumor-associated macrophages (TAMs) (Fig. 1b, c).

The IHC double staining studies documented strong co-localization of PZ and FX as well ZPI and FX in association with cancer cells (Fig. 1e, f). Additionally, co-localization of ZPI and PZ was observed in gastric cancer cells (Fig. 1g).

**Fig. 2:**
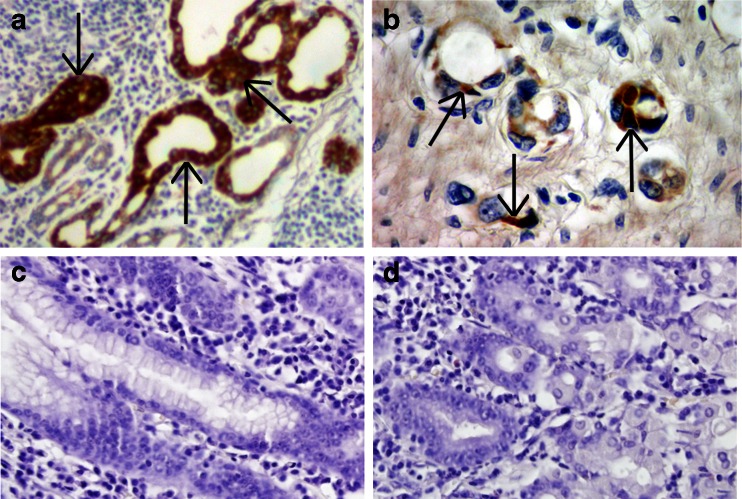
The in situ hybridization technique employed specific molecular probes directed to protein Z mRNA (**a**) and protein Z-dependent protease inhibitor mRNA (**b**). *Dark brown* staining (*solid arrows*) for the proteins in tumor cells of gastric cancer indicating constitutive synthesis of the proteins in these cells. No staining for PZ mRNA (**c**) or ZPI mRNA (**d**) was observed in normal gastric tissue. Hematoxylin counterstain, original magnification ×200 (**a**, **c**, **d**) and x400 (**b**)

The presence of ZPI and PZ mRNA were observed in neoplastic cells of gastric cancer (Fig. 2a, b). Neither PZ nor ZPI mRNA expressions were observed in normal gastric tissue (Fig. 2c, d).

## Discussion

Activation of FX is thought to be an important step in blood coagulation activation in malignancy [3–5]. A plethora of coagulation inhibitory mechanisms (among them PZ/ZPI system) are recognized to counterbalance the activated coagulation cascade [8, 9, 11, 14, 15]. The present study revealed the presence of PZ and ZPI in association with ECs lining vessels that supply nutrients to the gastric cancer, suggesting that the PZ-ZPI complex may contribute to limiting the rate of intravascular thrombin generation. Similarly, in non-small-cell lung cancer (NSCLC) tissue, both ZP and ZPI in association with ECs were demonstrated, whereas the presence of FX and PZ but not ZPI was revealed in colon cancer tissue [27, 28]. Contrary to previously published reports that human ECs are capable of synthesizing PZ [29, 30] and the presence of mRNAs encoding ZP or ZPI was demonstrated in colon cancer tissue [27], neither PZ nor ZPI mRNAs were observed in association with ECs in the present study. This suggests a blood-borne origin of both proteins in this localization.

To date, the evidence on the impact of ZPI/PZ on blood coagulation in cancer is vague. Of particular interest, the intron F G79A polymorphism of the PZ gene in cancer patients did not result in any coagulation abnormalities [31]. Furthermore, lower PZ plasma levels in cancer patients in comparison to a control group were reported, and decreasing PZ concentrations coincident with cancer progression were observed [32]. Interestingly, methylome analysis and integrative profiling of human hepatocellular carcinoma identified PZ as a tumor-suppressor gene [33]. Of note, decreased blood levels of PZ in acute leukemia and acute lymphoblastic leukemia patients were demonstrated, which correlated with increased episodes of bleeding in the latter subgroup of the patients [34].

It is a well-known notion that blood coagulation activation also proceeds extravascularly in tumor tissue [5]. Interestingly, tissue factor-dependent coagulation in human gastric cancer tissue was reported [2]. The authors documented the presence of cross-linked fibrin, the final product of blood coagulation, at gastric cancer sites [2]. Furthermore, they observed the expression of F1 + 2, which is a by-product formed during thrombin generation from the parent molecule, prothrombin [8, 24]. This indicator of extravascular blood coagulation in cancer tissue was found in association with gastric cancer cells. This was of special interest because our ABC IHC studies revealed the presence of both proteins (PZ and ZPI) and FX in gastric cancer cells. Furthermore, the double staining procedures revealed strict co-localization of PZ/ZPI system components, along with the expression of FX in gastric cancer cells in loco. Similar results were observed in colon cancer and NSCLC [27, 28]. Recently, Vasse [17] proposed the hypothesis that the major role of the PZ/ZPI system is to avoid the local tissue deposition of fibrin. However, this seems not to be the case in gastric cancer tissue. The presence of F1 + 2 at the tumor site suggests that the PZ/ZPI regulatory mechanism is insufficient at fully inhibiting blood activation in the strict vicinity of cancer cells. However, it should be recognized that the complex formed between ZPI and FXa is less stable than other serpin-protease complexes, and that the PZ/ZPI complex is unable to completely neutralize FXa catalytic activity under normal conditions [17]. Thus, our findings raise questions on the potential role of the PZ/ZPI system in this localization. Various non-hemostatic processes contributing to cancer progression were recognized to be triggered by blood coagulation inhibitors (reviewed in details elsewhere) [7]. Of note, our study demonstrated mRNAs encoding PZ and ZPI synthesis (apart from respective proteins expression) in gastric cancer cells, pointing to the constitutive expression of PZ and ZPI in gastric cancer cells. Similar findings were observed in human breast and colon cancer tissues [22, 23, 27]. Pancreatic endocrine cancers as well as enterochromaffin and neuroendocrine carcinoma cells were characterized by overexpression of ZPI mRNA in both primary and metastatic tumors [35, 36]. However, in lymphoblasts obtained from children suffering from acute lymphoblastic leukemia, no staining of PZ mRNA was observed, although some levels of ZPI mRNA were detected [17]. Noteworthy information was reported concerning the role of PZ and ZPI in obstetrics and inflammation, which suggests the potential function of the above inhibitors not only in the regulation of blood coagulation but also in local biological processes [17, 22, 23, 37]. Of interest is also the presence of the examined inhibitors in TAMs, which is in accordance with our previous findings in breast and colon cancer as well as in NSCLC tissues [22, 23, 27, 28].

Since the study does not provide insight into the pathophysiological mechanisms, further investigation is required.

## Conclusions

The co-localization of PZ/ZPI and FX in association with cancer cells in gastric cancer tissue indicates that the proteins may play a role in the anticoagulant events at the tumor tissue.
